# *In vitro* Characterization of Biofilm Formation in *Prevotella* Species

**DOI:** 10.3389/froh.2021.724194

**Published:** 2021-10-08

**Authors:** Shurooq Zakariya Albaghdadi, Jenan Bader Altaher, Hana Drobiova, Radhika G. Bhardwaj, Maribasappa Karched

**Affiliations:** Oral Microbiology Research Laboratory, Department of Bioclinical Sciences, Faculty of Dentistry, Health Sciences Center, Kuwait University, Jabriya, Kuwait

**Keywords:** biofilm, *Prevotella*, anaerobic, anti-biofilm, oral infections

## Abstract

**Background:** Periodontitis, a chronic inflammatory oral infection is the outcome of disturbances in the homeostasis of the oral biofilm microbiota. A number of studies have found the occurrence of *Prevotella* species in elevated levels in periodontitis compared to healthy subjects. Even though different aspects of *Prevotella* as part of oral biofilm have been studied, *in vitro* biofilms formed by these species have not been characterized systematically. The objective of this study was to characterize biofilms formed by several *Prevotella* species and further to assess biofilm inhibition and detachment of preformed biofilms.

**Methods:** Biofilms were grown in 24-well plates containing brucella broth in anaerobic conditions for 3 days, and were quantified using crystal violet staining. Images of SYTO 9 Green fluorescent stained biofilms were captured using confocal microscopy. Biofilm inhibition and detachment by proteinase and DNase I was tested. The biochemical characterization included quantification of proteins and DNA in the biofilms and biofilm-supernatants.

**Results:** *Prevotella loescheii, Prevotella oralis* and *Prevotella nigrescens* showed highest biofilm formation. *P. nigrescens* formed significantly higher amounts of biofilms than *P. loescheii* (*P* = 0.005) and *P. oralis* (*P* = 0.0013). Inhibition of biofilm formation was significant only in the case of *P. oralis* when treated with proteinase (*P* = 0.037), whereas with DNase I treatment, the inhibition was not significant (*P* = 0.531). Overall, proteinase was more effective in biofilm detachment than DNase I. Protein and DNA content were higher in biofilm than the supernatant with the highest amounts found in *P. nigrescens* biofilm and supernatants. *P. oralis* biofilms appeared to secrete large amounts of proteins extracellularly into the biofilm-supernatants.

**Conclusion:** Significant differences among *Prevotella* species to form biofilms may imply their variable abilities to get integrated into oral biofilm communities. Of the species that were able to grow as biofilms, DNase I and proteinase inhibited the biofilm growth or were able to cause biofilm detachment.

## Introduction

Bacterial biofilms are complex, highly organized functional communities embedded in an organic matrix known as extracellular polymeric substance (EPS) [1, 2]. Biofilms play an important role in medicine in general as they are a common cause of nosocomial infections, which are a leading cause of death [3]. Moreover, they are difficult to treat and handle due to their increased resistance to antibiotics, making them a challenge in the medical field.

The wide spread distribution and easy accessibility of oral biofilms render them a suitable model system for studying bacterial adhesion, biofilm development, and antibiotic resistance. Dental plaques are structurally and functionally organized biofilms, which develop in an ordered fashion and contain diverse microbial communities. The microorganisms in these biofilms tightly bind to each other and to the solid surface of the tooth by means of the organic extracellular matrix.

Oral biofilms generally comprise of commensal oral microbiota. However, disturbances in the homeostasis of the microbiota can lead to opportunistic oral infections such as dental caries and periodontitis [4, 5].

Periodontitis is a chronic inflammatory disease characterized by severe inflammation of the periodontal tissue, progressive destruction of ligament fibers, as well as alveolar bone loss [6]. The disease is primarily related to chronic plaque accumulation in a susceptible host. Major bacterial species implicated in periodontitis are *Aggregatibacter actinomycetemcomitans, Porphyromonas gingivalis, Tannerella forsythia*, and *Treponema denticola* [7]. The advent of high-throughput DNA sequencing technologies during the last decade have shown that the microbiota associated with periodontal disease is highly complex [8, 9]. A number of studies have found the occurrence of *Prevotella* species in elevated levels in periodontitis compared to healthy subjects [4, 10]. Further, presence of *Prevotella* has been positively correlated with clinical attachment loss [11], bleeding on probing [12], and periodontal inflammation [13]. *Prevotella* species are Gram-negative, non-motile, rod-shaped, singular cells that require strict anaerobic conditions for growth. In addition to oral infections, *Prevotella* species have been found in non-oral infections including abscesses, bacteremia, wound infections, bite infections, and genital tract infections.

Biofilm formation may be considered an important virulence trait of bacteria. For *Prevotella* species to survive in a complex and competitive oral environment, it is imperative that they can adhere to surfaces and integrate into oral biofilm. Previously, even though different aspects of *Prevotella* as part of oral biofilm have been studied [14, 15], *in vitro* biofilms formed by these species have not been characterized systematically. In clinically important systemic infections such as cystic fibrosis, proteinase treatment of the biofilms not only degrades proteins in the matrix, but also releases extracellular DNA (eDNA), which is targeted by using DNase I [16, 17]. Similar biofilm-modification approaches for oral biofilms could help in the development of newer treatment strategies for oral infections. Our aim was to characterize biofilms formed by several *Prevotella* species. We employed various methods to analyze biofilm contents, and then to investigate inhibition of biofilm formation and detachment of preformed biofilms.

## Methods

### Bacterial Strains and Culture Conditions

*Prevotella intermedia* ATCC 25611, *Prevotella nigrescens* ATCC 33563, *Prevotella loescheii* CCUG 5914, *Prevotella melaninogenica* CCUG 4944, *Prevotella oris* CCUG 15405, *Prevotella oralis* CCUG 15408, *Prevotella pallens* CCUG 39484, *Prevotella oulorum* CCUG 54769 were cultured on brucella blood agar containing 5% sheep blood for 3–4 days at 37°C in anaerobic atmosphere (10% H_2_, 5% CO_2_, 85% N_2_) using Anoxomat™ Mark II anaerobic gas filling system (Mart Microbiology, Netherlands). Prior to using the cultures in experiments, each strain was observed under a stereo microscope for confirming the colony morphology and to ensure that plates were devoid of contaminant growth.

### Biofilm Culture

Bacterial strains were cultured as described above. After that, a loopful of colonies from each strain was inoculated into the wells of a 24-well cell culture plate containing 1 ml brucella broth in each well. Wells with broth but no bacteria served as a negative control. The plates were incubated in anaerobic conditions as above for 3 days. At the end of incubation, supernatant broth was aspirated and the biofilms were washed once with sterile phosphate buffered saline (PBS) to remove unattached bacteria. Attached bacterial cells were fixed with methanol. The biofilms were subjected to quantification by various methods as below.

### Biofilm Quantification Assay

Biofilms were washed and air-dried and stained using 0.1% crystal violet stain (Sigma) [18]. First, biofilms were fixed by incubating in 1 ml of methanol per well for 15 min at room temperature. After that, the methanol was removed and the plate was dried for 45 min at room temperature. One ml of 0.1% aqueous crystal violet stain was added to each well and the plates were incubated for 20 min at room temperature. Finally, the wells were washed 5 times with tap water ensuring the complete removal of stain traces, dried and destained by shaking for 5 min with 500 μl/well of 33% acetic acid. Absorbance of stained acetic acid was measured at 590 nm (iMark^TM^ Microplate Absorbance Reader, Hercules, California, USA).

### Confocal Microscopy of Biofilms

For confocal imaging of the biofilms, all bacterial strains were grown as monospecies biofilms on Millicell^®^ EZ slides (EMD Millipore, Burlington, Massachussetts, USA) with detachable wells. After incubating for 3 days, supernatant broth was removed and the biofilms were washed one time with 1 ml sterile PBS to remove unbound and loosely attached bacteria. The biofilms were then fixed by adding 1 ml 4% freshly prepared paraformaldehyde in PBS and incubated for 30 min at room temperature. After removing the fixative solution, the biofilms were washed again as above. The biofilms were stained by directly adding SYTO^®^ 9 Green Fluorescent Nucleic Acid Stain (3.34 mM in DMSO) (Molecular Probes, Eugene, Oregon, USA) on to the biofilms. After a 15-min incubation in dark the staining solution was removed from the wells and the biofilms were washed as above. The polypropylene wells were detached by breaking the tabs and the slides were air-dried. A drop of mounting oil (Invitrogen) was placed on the biofilms and a cover glass placed. The stained biofilms were analyzed by confocal laser scanning microscopy (LSM 700, Carl Zeiss, Oberkochen, Germany).

### Biofilm Inhibition Assay

Biofilm cultures were set up as described above. Stock solutions of proteinase and DNase I were prepared in sterile distilled water and 150 mM NaCl, respectively. In separate experiments, DNAse from bovine pancreas (EC number 3.1.21.1, Sigma) and Proteinase from *Aspergillus melleus* (EC number 232-642-4, Sigma) were added into broth at a concentration of 10, 25, and 50 μg/ml. The plates were incubated anaerobically and biofilm formation was quantified by crystal violet staining method as mentioned above.

### Biofilm Detachment Assay

*Prevotella* biofilms were cultured in 24-well plates as described above. Wells were washed with sterile PBS once to remove loosely adherent cells. Wells were then filled with 500 μl of proteinase from *Aspergillus melleus* (10, 25, and 50 μg/ml) or DNase I from bovine pancreas (10, 25, and 50 μg/ml). Wells filled with water alone were used as negative controls. After incubating for 1 h at room temperature, wells were washed to remove loosely adherent cells and biofilms were quantified by crystal violet staining method as mentioned above.

### Quantification of Protein and DNA Contents of Biofilms

Protein and DNA contents of the biofilms and biofilm-supernatants were quantified. Biofilms were grown in 100-mm cell culture polystyrene dishes in the same culture conditions as described above. The biofilms were washed with distilled water and the biofilm mass were removed with a cell scraper and collected in 5 ml of 0.9% NaCl. The biofilm suspension was homogenized by vigorous vortexing and divided into two parts. One half of the suspension was filtered through a 0.2 μm PES filter (EMD Millipore). Protein content of the filtered suspension (biofilm) and of the biofilm-supernatant was determined by a bicinchoninic acid assay (Pierce, Waltham, Massachussetts, USA). Bovine serum albumin was used for constructing a standard curve. DNA from the biofilms and biofilm-supernatants was purified using DNeasy DNA Purification Kit (Qiagen, Hilden, Germany) according to manufacturer's protocol. Quantities of the purified DNA were determined spectrophotometrically by measuring A260 on a Nanodrop instrument.

### Statistics

Data were not normally distributed as determined by Skewness and Kurtosis values, Shapiro Wilkins *P*-values and histograms. Therefore, quantitative differences between the groups were compared by Mann Whitney U test. Statistical analysis was done using SPSS vs. 22 for Windows. All experiments were performed at least 3 times and in each experiment, biological and technical replicates were included. *P*-values lower than 0.05 are regarded statistically significant.

## Results

### Biofilm Formation by *Prevotella* spp.

**Figure 1:**
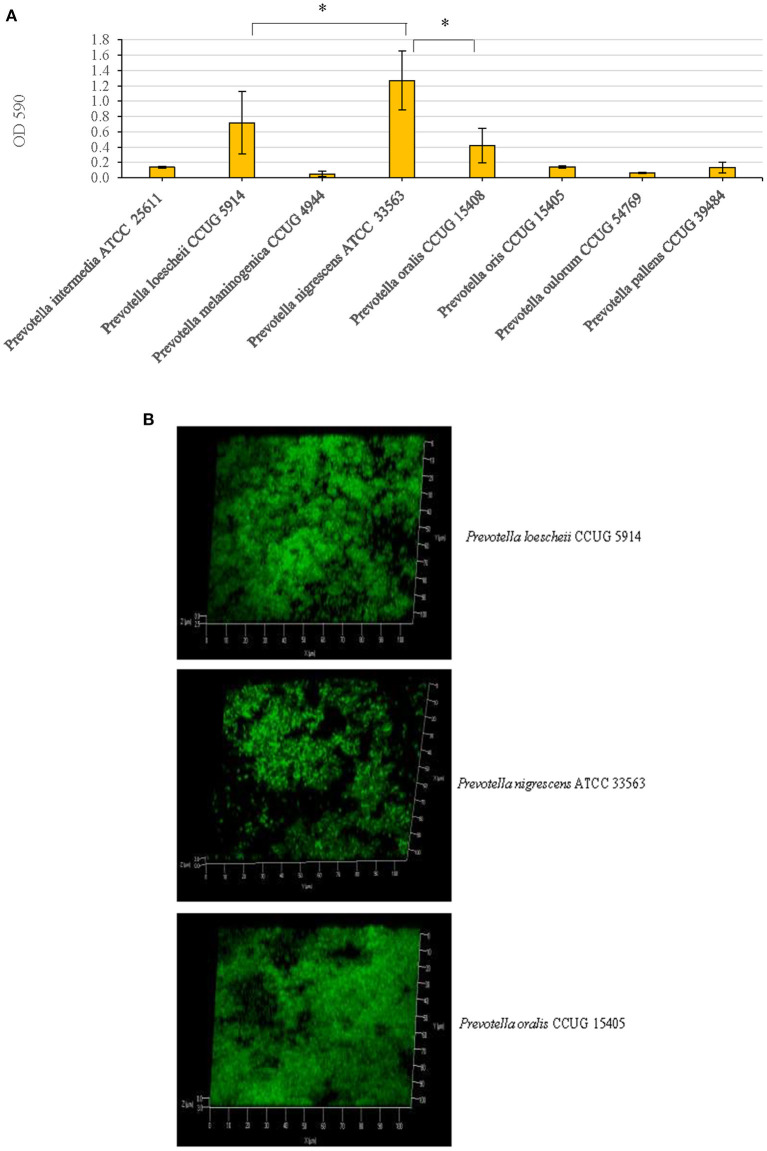
**(A)** Biofilm forming abilities of eight *Prevotella* species. After growing static biofilm cultures for 3 days, a standard crystal violet staining technique was used for the quantification of the biofilm mass. Optical densities of the crystal violet solutions post-biofilm staining were measured at 590 nm. ^*^P < 0.005, Mann Whitney. **(B)** Confocal microscopy images of the biofilms. *P. loescheii, P. nigrescens*, and *P. oralis* biofilms which were cultured on glass chambers and stained with SYTO 9 dye. The images show *x*-*y*-*z* projections for a 3D view.

Of the eight *Prevotella* spp. that were tested for their biofilm forming abilities, *P. oralis, P. loeschii* and *P. nigrescens* showed the highest biofilm formation in an increasing order. Therefore, these three species were further studied for biofilm characterization. *P. nigrescens* exhibited significantly higher amount of biofilm than all other species including *P. loescheii* (*P* = 0.005) and *P. oralis* (*P* = 0.0013) (Figure 1A).

Confocal microscopic images revealed that the three species, *P. oralis, P. loeschii*, and *P. nigrescens* formed dense and thick biofilms. The 3D images showed that the cells in the biofilms were arranged as clusters and isolated single cells (Figure 1B).

### Biofilm Inhibition and Detachment

To determine how the addition of proteinase and DNase I influences the biofilm growth, the two enzymes were added at the beginning when the biofilm cultures were initiated. Whether proteinase and DNase I cause detachment of preformed biofilms was studied by treating the biofilms with different concentrations of the two enzymes.

#### Effect of Proteinase on Biofilm Inhibition and Detachment

**Figure 2:**
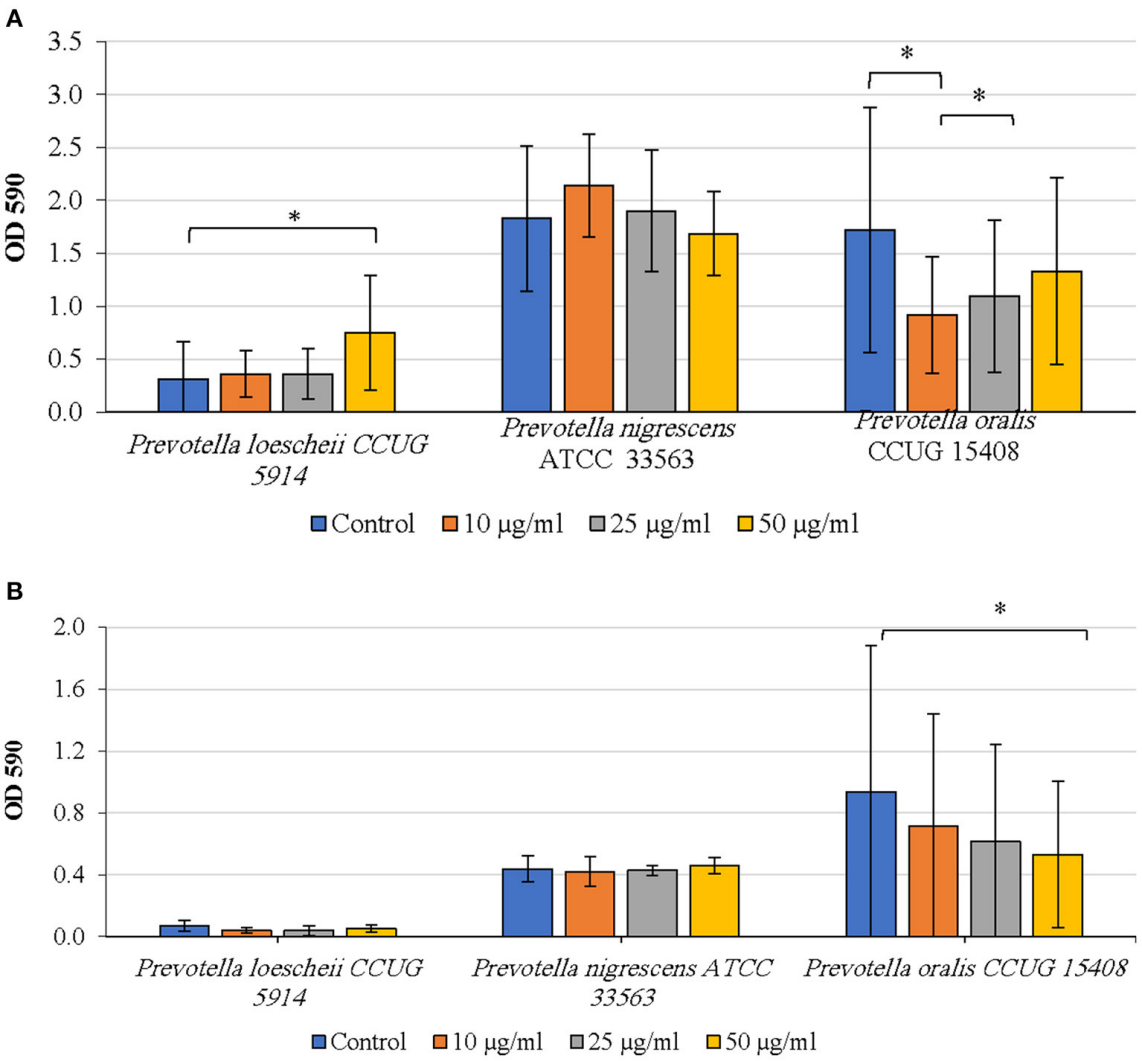
**(A)** Inhibition of biofilm formation by proteinase. The effect of proteinase treatment on the initiation and development of biofilms was studied. Different concentrations of proteinase were added to the biofilm culture medium at the same time as the inoculum and incubated as described in the methods section. Biofilm mass was quantified using crystal violet technique. ^*^P < 0.005 Mann Whitney. **(B)** Detachment of preformed biofilms by proteinase. The effect of proteinase treatment on the preformed biofilms was studied. Different concentrations of proteinase were added to the biofilm culture medium at the same time as the inoculum and incubated as described in the methods section. Biofilm mass was quantified using crystal violet technique. ^*^P < 0.005, Mann Whitney.

The effect of proteinase treatment on the biofilm growth varied among species. In *P. oralis*, biofilm formation was inhibited at all concentrations of proteinase when compared to the untreated control; however, the inhibition was significant only at 10 μg/ml (*P* = 0.037) (Figure 2A). Further, increasing concentrations of proteinase did not seem to inhibit biofilm formation in a concentration dependent fashion; rather the inhibitory effect decreased with increasing proteinase concentrations. Proteinase treatment did not show an inhibitory activity on *P. loescheii* and *P. nigrescens*.

Detachment of *P. oralis* biofilm increased with increasing concentrations of proteinase (Figure 2B). The decrease in the biofilm mass of *P. oralis* following proteinase treatment was significant compared to the untreated control (*P* = 0.0038). No reduction in the biofilm amounts of *P. loescheii* or *P. nigrescens* was seen.

#### Effect DNase I on Biofilm Inhibition and Detachment

**Figure 3:**
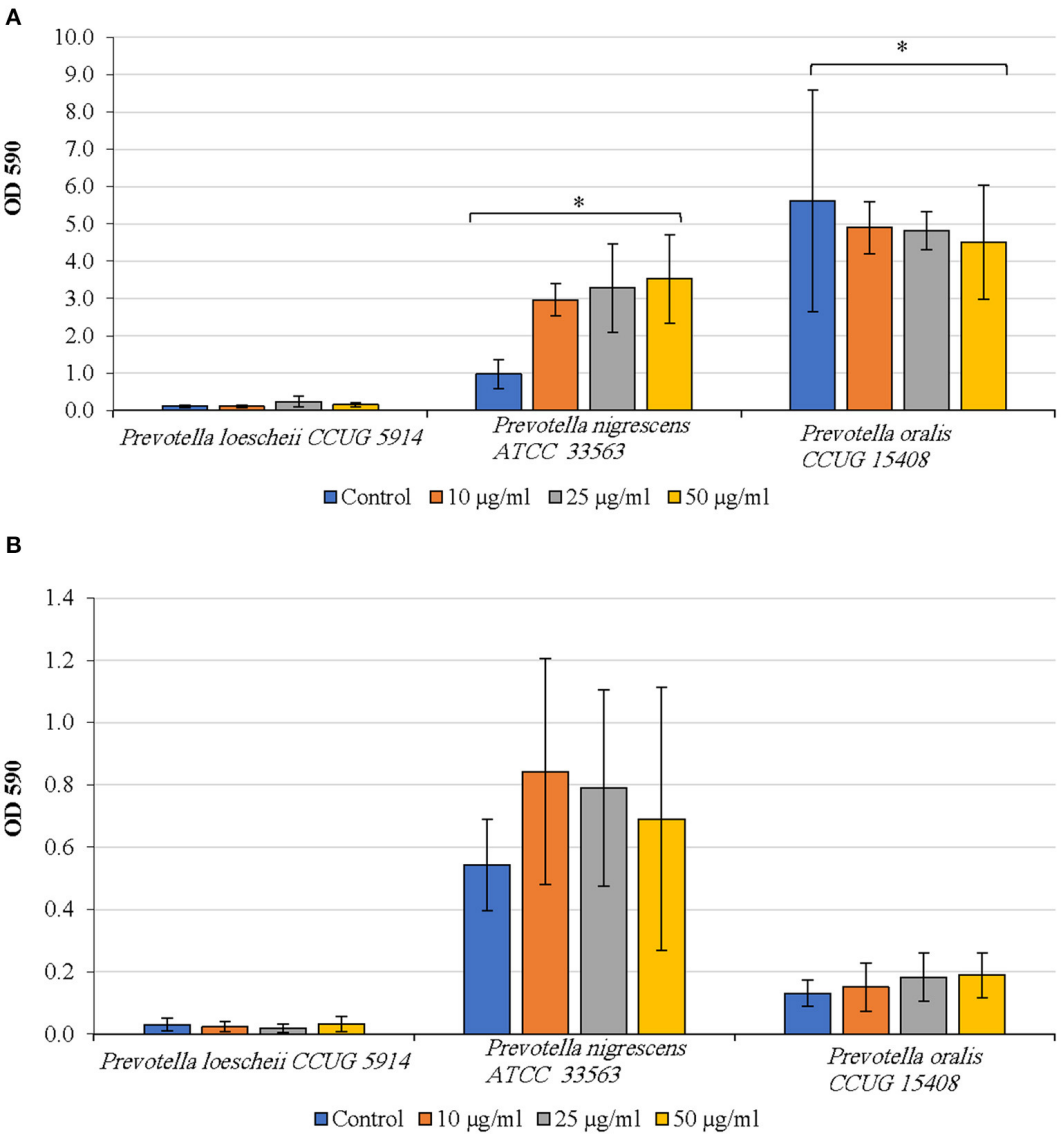
**(A)** Inhibition of biofilm formation by DNase I. The effect of DNase treatment on the initiation and development of biofilms was studied. Different concentrations of DNase were added to the biofilm culture medium at the same time as the inoculum and incubated as described in the methods section. Biofilm mass was quantified using crystal violet technique. ^*^P < 0.005, Mann Whitney. **(B)** Detachment of preformed biofilms by DNase I. The effect of DNase treatment on the preformed biofilms was studied. Different concentrations of the enzyme were added to the biofilm culture medium at the same time as the inoculum and incubated as described in the methods section. Biofilm mass was quantified using crystal violet technique. ^*^P < 0.005, Mann Whitney.

*P. oralis* biofilms showed a concentration dependent decrease in biofilm quantities upon treatment with DNase I (Figure 3A). However, even at the highest concentration of DNase I (50 μg/ml), the inhibition was not statistically significant compared to the untreated control (*P* = 0.531). While *P. loescheii* formed poor biofilms in these experiments, DNase I treatment seemed to enhance biofilm formation in *P. nigrescens*. Increasing concentrations of DNase I resulted in a significant increase of biofilm amounts of *P. nigrescens* (*P* = 0.009).

As seen in Figure 3B, DNase I treatment did not result in biofilm detachment of any species. *P. oralis* biofilms appeared to be increasing in quantities as the DNase I concentration increased. *P. nigrescens* formed higher amounts of biofilms upon DNase I treatment than the untreated control. *P. loescheii* showed poor biofilm growth in untreated and treated biofilms.

### Biochemical Analysis of Biofilms

As seen in Table 1, mean (SD) biofilm protein concentration of *P. nigrescens*, which was highest of all, was 1,745 (271) μg per ml, followed by *P. oralis*, 1,416 (188) and *P. loescheii*, 1,070 (72) μg per ml. In the case of biofilm-supernatants, *P. oralis* exhibited highest protein amounts of 1,153 (564) μg per ml. Overall, the protein concentrations in biofilm mass were significantly higher than those of supernatants (*P* < 0.05). Highest concentrations of DNA were detected in *P. nigrescens* biofilm. Similar to protein amounts, biofilms showed a higher DNA content than the supernatants.

**Table 1:** Biochemical characterization of the *Prevotella* biofilms.

	**Mean (SD)**			
	**DNA (μg/ml)**		**Protein (μg/ml)**	
**Species**	**Biofilm**	**Supernatant**	**Biofilm**	**Supernatant**
*P. loescheii*	22.5 (8.8)	10.6 (7.8)	1,070 (72)	323 (177)
*P. nigrescens*	337.4 (131.6)	5.3 (0.9)	1,745 (271)	561 (142)
*P. oralis*	101.6 (23.6)	10.5 (2.5)	1,416 (188)	1,153 (564)

## Discussion

In this study, using a simple crystal violet staining method, we tested eight *Prevotella* species for their ability to form biofilms on a polystyrene surface. We found that only three of the eight species, *P. oralis, P. loeshii*, and *P. nigrescens*, demonstrated ability to form biofilms.

Biofilm formation is generally regarded as a virulence strategy of bacteria. Biofilm formation renders the bacteria more resistant to antibacterial treatment including antibiotics, proteolytic enzymes and DNases compared to their planktonic counterparts, because the extracellular matrix of the biofilm functions as a critical barrier that limits the efficacy of antibacterial treatment agents. Biofilm matrix contains abundant protein and eDNA. Proteinases catalyze the cleavage of peptide bonds and lead to protein degradation [19]. Proteinases can be either intracellular or extracellular in nature. The extracellular proteases are less selective in their substrate recognition and can cleave both self and non-self-molecules with equal efficiency. Moreover, eDNA is essential for biofilm formation in many species [20]. Enzymatic disruption of this DNA specifically inhibits newly forming and established biofilms. DNase I degrades the eDNA present in the matrix liberating bacteria from the biofilm to supernatant fractions, making the matrix weak and susceptible and potentiates the activity of antimicrobial agents [21, 22].

We used Proteinase-K and DNase I, two matrix degrading enzymes to study their potential to inhibit biofilm formation as well as to detach preformed biofilms. In our findings, proteinase and DNase I enzymes showed an inhibitory effect on the biofilm formation of *P. oralis* but DNase I treatment did not result in the detachment of biofilms. Moreover, the results on biofilm inhibition and detachment were unclear in the case of *P. loeschii* and *P. nigrescens*. Overall, no clear inhibition or detachment could be seen. Further, concentration dependent proteinase increase did not seem to inhibit biofilm formation and no inhibitory activity or reduction in biofilm amounts in *P. loescheii* and *P. nigrescens* was noticed. It is possible that the enzymes were inactivated by the extracellular components in the biofilms of these species [23].

Recently, Karygianni et al. [24] noticed that the enzymes DNase I and Proteinase interference with composition and integrity of the biofilms. In the above study, confocal images revealed a loss of biofilm integrity and a favored growth of streptococci among the biofilm organisms upon combined application of DNase I and proteinase K. Significant suppression in the growth of *Actinomyces oris, Fusobacterium nucleatum, Streptococcus mutans, Streptococcus oralis*, and *Candida albicans* by DNase I was noticed. However, Proteinase K treatment induced significant increase in *S. mutans* and *S. oralis* colony forming units (CFU), whereas *C. albicans* and *V. dispar* showed lower CFU. No negative impact on total bacterial counts by DNase I and proteinase K treatment implies that both enzymes interact only with eDNA and extracellular matrix proteins, without penetrating the intact membranes of the microbial cells, thereby interfering solely with the structural integrity of the biofilms. In dental procedures such as root canal disinfection, a variety of antimicrobial disinfectant irrigants have been used [25, 26]. Given the limited efficacy of antimicrobials in penetrating mature biofilms, application of enzymes such as DNase I and proteinase K could degrade EPS thereby making the biofilm permeable to antimicrobials. Such a combination of antimicrobials and enzymes has been investigated earlier [22, 27].

Protein and eDNA content of the biofilms play a role in the formation of biofilm matrix and resistance to anti-biofilm strategies [28]. In our study, the protein and DNA contents varied between the species. *P. nigrescens* biofilms showed highest quantities of DNA and protein. In the biofilm supernatants, the DNA concentrations were very low in all three species. Biofilm matrix composition, including the protein and extracellular DNA, varies among species and is also influenced by the composition of the biofilm growth medium [29].

A major limitation of this study is that the *Prevotella* biofilms could have been characterized more comprehensively. For example, scanning electron microscopy of the biofilms, profiling the expression of biofilm-associated genes, and characterization of EPS using techniques such as HPLC would have yielded more understanding of *Prevotella* biofilms. Further, treating the biofilms with antibiotics relevant to oral infections, and use of multispecies biofilms comprising different *Prevotella* species may provide more insightful information about the biofilm lifestyle of these species. In our upcoming study, we may attempt further comprehensive characterization of *Prevotella* biofilms, including but not limited to finding novel biofilm-essential genes using random transposon mutagenesis.

## Conclusion

*P. loescheii, P. nigrescens*, and *P. oralis* are important species belonging to the genus *Prevotella* in particular but also are important members of human oral microbiota. Biofilm inhibition experiments showed that proteinase and DNase I enzymes have an inhibitory effect on biofilm of *P. oralis*. Biofilm detachment experiments showed proteinase does have an effect on the biofilm of *P. oralis*. Our results showing differential protein and DNA content among species, and differences in biofilm inhibition and detachment, suggest that *Prevotella* species might possess strategies for surviving as biofilm communities and that their inhibition by proteinases or DNases could be exploited in treatment strategies.

## Data Availability Statement

The original contributions presented in the study are included in the article/supplementary files, further inquiries can be directed to the corresponding author/s.

## Author Contributions

SA performed experiments, organized the data, and wrote the manuscript. JA performed experiments, organized the data, and critically read the manuscript. HD supervised experiments by SA and JA, analyzed data, and critically read the manuscript. RB helped in planning the experiments, analyzed data, and wrote the manuscript. MK conceptualized the study, planned experiments, analyzed and interpreted data, and wrote the manuscript. All authors contributed to the article and approved the submitted version.

## Funding

This study was supported by Kuwait University Grant SRUL 01/14.

## Conflict of Interest

The authors declare that the research was conducted in the absence of any commercial or financial relationships that could be construed as a potential conflict of interest.

## Publisher's Note

All claims expressed in this article are solely those of the authors and do not necessarily represent those of their affiliated organizations, or those of the publisher, the editors and the reviewers. Any product that may be evaluated in this article, or claim that may be made by its manufacturer, is not guaranteed or endorsed by the publisher.

## References

[1] Branda SS Vik S Friedman L Kolter R. Biofilms: the matrix revisited. Trends Microbiol. (2005) 13:20–6. 10.1016/j.tim.2004.11.006 15639628

[2] Flemming HC Wingender J. The biofilm matrix. Nat Rev Microbiol. (2010) 8:623–33. 10.1038/nrmicro2415 20676145

[3] Bryers JD. Medical biofilms. Biotechnol Bioeng. (2008) 100:1–18. 10.1002/bit.21838 18366134 PMC2706312

[4] Jenkinson HF Lamont RJ. Oral microbial communities in sickness and in health. Trends Microbiol. (2005) 13:589–95. 10.1016/j.tim.2005.09.006 16214341

[5] Ximenez-Fyvie LA Haffajee AD Socransky SS. Comparison of the microbiota of supra- and subgingival plaque in health and periodontitis. J Clin Periodontol. (2000) 27:648–57. 10.1034/j.1600-051x.2000.027009648.x 10983598

[6] Flemmig TF. Periodontitis. Ann Periodontol. (1999) 4:32–8. 10.1902/annals.1999.4.1.32 10863373

[7] Socransky SS Haffajee AD. The bacterial etiology of destructive periodontal disease: current concepts. J Periodontol. (1992) 63(4 Suppl.):322–31. 10.1902/jop.1992.63.4s.322 1573546

[8] Belstrom D Sembler-Moller ML Grande MA Kirkby N Cotton SL Paster BJ. Microbial profile comparisons of saliva, pooled and site-specific subgingival samples in periodontitis patients. PLoS ONE. (2017) 12:e0182992. 10.1371/journal.pone.0182992 28800622 PMC5553731

[9] Colombo AP Boches SK Cotton SL Goodson JM Kent R Haffajee AD. Comparisons of subgingival microbial profiles of refractory periodontitis, severe periodontitis, and periodontal health using the human oral microbe identification microarray. J Periodontol. (2009) 80:1421–32. 10.1902/jop.2009.090185 19722792 PMC3627366

[10] Fukui K Kato N Kato H Watanabe K Tatematsu N. Incidence of *Prevotella intermedia* and *Prevotella nigrescens* carriage among family members with subclinical periodontal disease. J Clin Microbiol. (1999) 37:3141–5. 10.1128/JCM.37.10.3141-3145.1999 10488167 PMC85513

[11] Dahlen G Claesson R Aberg CH Haubek D Johansson A Kwamin F. Subgingival bacteria in Ghanaian adolescents with or without progression of attachment loss. J Oral Microbiol. (2014) 6:1–6. 10.3402/jom.v6.23977 24834145 PMC4013489

[12] Joshi V Matthews C Aspiras M de Jager M Ward M Kumar P. Smoking decreases structural and functional resilience in the subgingival ecosystem. J Clin Periodontol. (2014) 41:1037–47. 10.1111/jcpe.12300 25139209

[13] Xie G Chain PS Lo CC Liu KL Gans J Merritt J. Community and gene composition of a human dental plaque microbiota obtained by metagenomic sequencing. Mol Oral Microbiol. (2010) 25:391–405. 10.1111/j.2041-1014.2010.00587.x 21040513 PMC2975940

[14] Moon JH Jang EY Shim KS Lee JY. *In vitro* effects of N-acetyl cysteine alone and in combination with antibiotics on *Prevotella intermedia*. J Microbiol. (2015) 53:321–9. 10.1007/s12275-015-4500-2 25935303

[15] Wakabayashi H Yamauchi K Kobayashi T Yaeshima T Iwatsuki K Yoshie H. Inhibitory effects of lactoferrin on growth and biofilm formation of *Porphyromonas gingivalis* and *Prevotella intermedia*. Antimicrob Agents Chemother. (2009) 53:3308–16. 10.1128/AAC.01688-08 19451301 PMC2715627

[16] Das T Simone M Ibugo AI Witting PK Manefield M Manos J. Glutathione enhances antibiotic efficiency and effectiveness of DNase I in disrupting *Pseudomonas aeruginosa* biofilms while also inhibiting pyocyanin activity, thus facilitating restoration of cell enzymatic activity, confluence and viability. Front Microbiol. (2017) 8:2429. 10.3389/fmicb.2017.02429 29312161 PMC5729223

[17] Wu J Xi C. Evaluation of different methods for extracting extracellular DNA from the biofilm matrix. Appl Environ Microbiol. (2009) 75:5390–5. 10.1128/AEM.00400-09 19561191 PMC2725481

[18] Peeters E Nelis HJ Coenye T. Comparison of multiple methods for quantification of microbial biofilms grown in microtiter plates. J Microbiol Methods. (2008) 72:157–65. 10.1016/j.mimet.2007.11.010 18155789

[19] Gilan I Sivan A. Effect of proteases on biofilm formation of the plastic-degrading actinomycete Rhodococcus ruber C208. FEMS Microbiol Lett. (2013) 342:18–23. 10.1111/1574-6968.12114 23448092

[20] Whitchurch CB Tolker-Nielsen T Ragas PC Mattick JS. Extracellular DNA required for bacterial biofilm formation. Science. (2002) 295:1487. 10.1126/science.295.5559.1487 11859186

[21] Nijland R Hall MJ Burgess JG. Dispersal of biofilms by secreted, matrix degrading, bacterial DNase. PLoS ONE. (2010) 5:e15668. 10.1371/journal.pone.0015668 21179489 PMC3001887

[22] Tetz GV Artemenko NK Tetz VV. Effect of DNase and antibiotics on biofilm characteristics. Antimicrob Agents Chemother. (2009) 53:1204–9. 10.1128/AAC.00471-08 19064900 PMC2650517

[23] Merritt JH Kadouri DE O'Toole GA. Growing and analyzing static biofilms. Curr Protoc Microbiol. (2005). 1–29. 10.1002/9780471729259.mc01b01s00 18770545 PMC4568995

[24] Karygianni L AT Thurnheer T. Combined DNase and proteinase treatment interferes with composition and structural integrity of multispecies oral biofilms. J Clin Med. (2020) 9:983. 10.3390/jcm9040983 32244784 PMC7231231

[25] Gonçalves LS Val Rodrigues RC Andrade Junior CV Soares RG Vettore MV. The effect of sodium hypochlorite and chlorhexidine as irrigant solutions for root canal disinfection: a systematic review of clinical trials. J Endod. (2016) 42:527–32. 10.1016/j.joen.2015.12.021 26852149

[26] Zehnder M. Root canal irrigants. J Endod. (2006) 32:389–98. 10.1016/j.joen.2005.09.014 16631834

[27] Suri R. The use of human deoxyribonuclease (rhDNase) in the management of cystic fibrosis. BioDrugs. (2005) 19:135–44. 10.2165/00063030-200519030-00001 15984899

[28] Fong JNC Yildiz FH. Biofilm matrix proteins. In: Ghannoum M Parsek M Whitley M Mukherjee PK editors. Microbial Biofilms. Washington, DC: ASM Press (2015). p. 201–22. 10.1128/9781555817466.ch10

[29] Ghannoum M Parsek M Whitley M Mukherjee PK. Microbial Biofilms. Washington, DC: ASM Press (2015). p. 404. 10.1128/9781555817466

